# Iatrogenic coronary artery dissection: detection, management, and prevention

**DOI:** 10.3389/fcvm.2026.1910377

**Published:** 2026-09-24

**Authors:** Yanyou Xie, Xia Liu, Kun Gong, Xiuxiu Fu, Kai Tan, Hui Yan, Cheng Zhao, Juan Gao

**Affiliations:** 1 Department of Cardiology, The Affiliated Hospital of Qingdao University, Qingdao University, Qingdao, China; 2 Department of Abdominal Ultrasonography, The Affiliated Hospital of Qingdao University, Qingdao, China; 3 Department of Echocardiography, The Affiliated Hospital of Qingdao University, Qingdao, China; 4 Outpatient Nursing Unit, The Affiliated Hospital of Qingdao University, Qingdao, China

**Keywords:** iatrogenic coronary artery dissection, imaging evaluation, intravascular imaging, percutaneous coronary intervention, procedural cardiac complication

## Abstract

Percutaneous coronary intervention (PCI) has become a cornerstone treatment for symptomatic coronary artery disease. Although generally safe and effective, PCI may still lead to serious complications such as coronary artery dissection or vessel perforation. Coronary artery dissection can arise spontaneously or be triggered by catheter-based procedures, the latter referred to as iatrogenic coronary artery dissection (ICAD). ICAD, while uncommon, carries significant procedural and prognostic implications; catheter-induced ostial dissection has been reported in approximately 0.09% of coronary angiographic procedures, while 30-day mortality reached 20.8% in a high-risk surgical cohort of patients with PCI-related coronary injuries. Given the increasing use of coronary interventions and the complexity of contemporary lesions, early recognition and accurate characterization of ICAD have become critical. Advances in intravascular imaging—particularly intravascular ultrasound and optical coherence tomography—have improved the ability to identify dissection morphology, understand underlying mechanisms, and guide real-time management. Furthermore, accumulating evidence highlights the role of patient characteristics, lesion anatomy, catheter/device interactions, and procedural techniques in shaping ICAD risk. This narrative review integrates current knowledge on the classification, imaging features, risk determinants, management strategies, and prevention of ICAD, providing a structured framework to support safer and more precise coronary intervention.

## Highlights

- ICAD is an uncommon but serious complication requiring systematic mechanistic understanding.
- Patient, lesion, device, and technique factors collectively determine ICAD risk.
- Imaging-defined dissection characteristics guide prognosis and inform management strategies.
- Future work should refine prevention strategies and validate imaging-guided treatment algorithms.

## Introduction

Coronary artery dissection, whether spontaneous or procedure-induced, arises from intimal tears or subintimal hematoma that can rapidly obstruct coronary blood flow and even cause occlusion of the lumen. Coronary artery dissection is mainly divided into spontaneous coronary artery dissection (SCAD) or iatrogenic coronary artery dissection (ICAD) (1–3). With the expanded use of plaque-modifying techniques, percutaneous coronary intervention (PCI)-related coronary dissection remains an important procedural complication, particularly in complex and heavily calcified lesions (4, 5); however, it cannot be entirely avoided. Early detection and well-chosen treatment strategies, such as PCI, surgery, or vigilant monitoring, can help prevent serious complications including ventricular dysfunction, cardiogenic shock, procedural myocardial infarction, or cardiac death (6). Although uncommon, ICAD is a potentially serious complication of PCI that requires prompt recognition and appropriate management (Figure 1).

**Figure 1:**
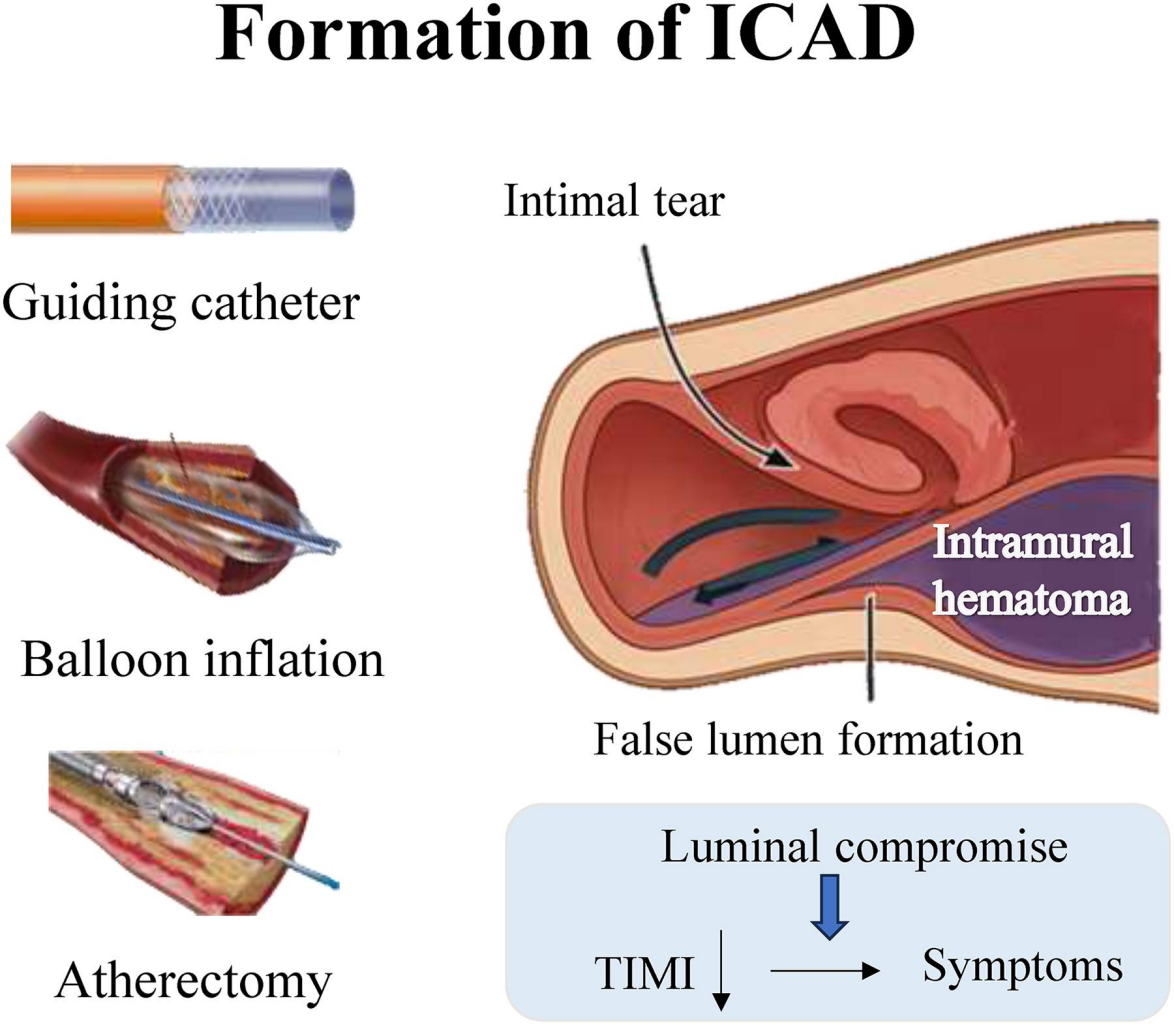
Mechanisms and formation of iatrogenic coronary artery dissection (ICAD). Catheter- and device-related mechanical injury, including guiding catheter manipulation, balloon inflation, and atherectomy, may cause intimal disruption, leading to intimal tear, false lumen formation, or intramural hematoma. These changes may compromise the true lumen, reduce Thrombolysis in Myocardial Infarction flow, and result in clinical symptoms. The figure was created by the authors using Microsoft PowerPoint.

Despite the increasing awareness of ICAD, current literature remains fragmented and lacks clinical guidance. Previous reviews have largely emphasized SCAD, while ICAD—despite being more commonly encountered in interventional practice—has received limited systematic evaluation. Most published evidence consists of isolated case reports or small observational studies, resulting in a lack of consensus on optimal diagnosis and management strategies (7–9). In particular, the decision-making process regarding conservative therapy versus stenting or emergent coronary artery bypass grafting (CABG) remains controversial. Additionally, although intravascular imaging modalities such as optical coherence tomography (OCT) and intravascular ultrasound (IVUS) have improved the characterization of ICAD, their roles have not been uniformly integrated into clinical practice.

Therefore, this review aims to provide an updated, clinically oriented summary of ICAD with a focus on its detection and interventional management. We highlight current diagnostic challenges, summarize procedural risk factors, and discuss evidence-based management and prevention strategies (Figure 2).

**Figure 2:**
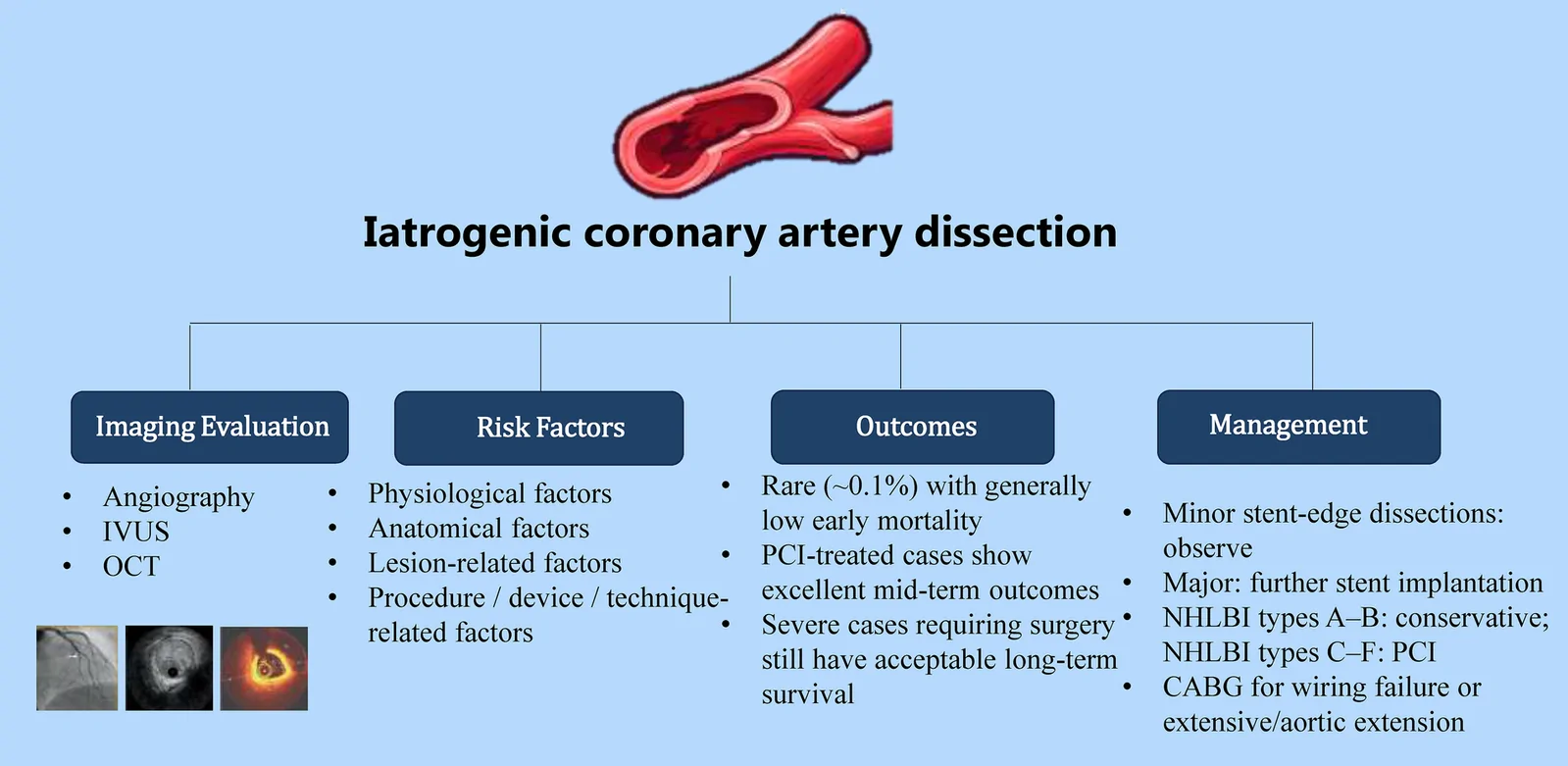
Overview of imaging, risk factors, outcomes, and management of iatrogenic coronary artery dissection (ICAD). ICAD can be evaluated using coronary angiography, intravascular ultrasound, and optical coherence tomography. Risk factors encompass physiological, anatomical, lesion-related, and procedure-, device-, or technique-related factors. Clinical outcomes vary according to dissection severity, while management ranges from conservative observation for minor or stable dissections to percutaneous coronary intervention or coronary artery bypass grafting for more severe cases. The figure was created by the authors using Microsoft PowerPoint.

## Literature search

An updated literature search was conducted in PubMed and Web of Science to identify relevant English-language publications on ICAD from database inception to 26 August 2026. The following search string was used in both databases: (“iatrogenic coronary artery dissection” OR “catheter-induced coronary artery dissection” OR “catheter induced coronary dissection” OR “iatrogenic aortocoronary dissection” OR “iatrogenic aorto-coronary dissection”). The search identified 99 records in PubMed and 130 records in Web of Science. After removal of 100 duplicate records, 129 unique records underwent title/abstract and full-text screening, and 97 records were retained as relevant to the narrative synthesis. Publications addressing the mechanisms, detection, classification, risk factors, prevention, management, or prognosis of ICAD in the context of coronary angiography (CAG) or PCI were considered relevant, whereas publications focused exclusively on spontaneous coronary artery dissection without relevance to procedure-induced dissection and records unrelated to ICAD were excluded. After duplicate removal, the retrieved records were screened for relevance to these predefined domains. Additional pertinent studies, particularly those addressing intravascular imaging, lesion characteristics, procedural devices and techniques, and therapeutic strategies, were identified through manual screening of the reference lists of retrieved articles and targeted supplementary literature searches. The 89 references ultimately cited were selected from these complementary sources based on their relevance to the narrative synthesis and to avoid redundant evidence. Given the narrative nature of this review, the search was intended to support a broad synthesis of the available evidence rather than a systematic or quantitative evidence synthesis.

## Classification and detection of ICAD

In this review, ICAD is broadly defined as any coronary artery dissection caused by diagnostic or interventional coronary procedures. For clarity, we distinguish catheter-induced dissection, which typically involves the coronary ostium and may extend into the aortic root, from device-induced dissection, including stent-edge and guidewire-related injury. At present, the primary classification of ICAD is based on CAG and intravascular imaging (IVI). Compared with IVI, CAG provides more limited visualization of subtle vessel-wall abnormalities and may miss smaller dissection flaps and intramural hematomas, with potentially catastrophic consequences; nevertheless, as the fundamental first-line examination, CAG plays an irreplaceable role in detecting ICAD. Below, we summarize the classification and characteristics of ICAD using CAG and IVI. Importantly, the imaging-based criteria discussed below should be interpreted according to the specific procedural context; in particular, OCT- and IVUS-derived morphological thresholds established for post-PCI stent-edge dissections should not be directly extrapolated to catheter-induced ostial or aortocoronary dissections.

### Classification and detection of ICAD by angiography

The intimal-tear classification system established by the National Heart, Lung, and Blood Institute (NHLBI), originating from the Coronary Angioplasty Registry, was introduced in the pre-stent era to describe angioplasty-related dissections based on their angiographic appearance (10). Dissection was graded based on the appearance on CAG as types A–F. Specifically, Type A represents minor radiolucent areas within the coronary lumen with minimal or no contrast persistence, indicating insignificant intimal injury. Type B is characterized by parallel tracts or a double lumen separated by a radiolucent area, again without contrast persistence after dye clearance. Type C denotes contrast outside the coronary lumen with persistent staining, implying an extraluminal extension of the tear. Type D dissections exhibit a spiral luminal filling defect, often accompanied by extensive contrast staining of the vessel wall. Type E refers to new, persistent filling defects, typically related to intraluminal thrombus formation, while Type F represents total occlusion of the vessel with absent distal antegrade flow. In some cases, a coronary artery dissection may propagate retrogradely and involve the ascending aorta (11, 12).

Among these, types A and B are usually benign and self-limiting, whereas types C–F are associated with impaired blood flow, higher rates of acute vessel closure, and a greater need for emergency revascularization. This morphological grading system, though developed before the stent era, remains an important historical and conceptual framework for understanding angioplasty-related coronary dissection and its clinical consequences.

### Classification and detection of ICAD by IVUS

Over the past three decades, IVUS has evolved as a valuable imaging modality for guiding PCI and for detecting procedure-related coronary dissections with high spatial resolution. IVUS can reveal subtle vessel wall injuries that are frequently underestimated or even invisible on CAG. Previous studies have reported that IVUS can identify stent edge dissections in approximately 9.2% of drug-eluting stent (DES) implantations, underscoring its superior sensitivity compared with angiographic assessment (13). Moreover, parameters such as residual plaque eccentricity, lumen-to-stent edge area ratio, and stent edge symmetry have been demonstrated to predict the occurrence of stent edge dissections (13). Based on the IVUS-derived morphological characteristics, coronary dissections can be classified into four major types: (1) intimal dissection, defined as a tear limited to the intimal layer with minimal disruption of the media; (2) medial dissection, involving deeper extension of the tear into the medial layer and often associated with a visible false lumen; (3) intramural hematoma, characterized by blood accumulation within the media without a clear entry point, leading to crescent-shaped vessel wall thickening; and (4) extramedial injury, representing the most extensive form of vessel wall damage with disruption beyond the external elastic membrane into the adventitia (3).

Compared with the angiography-based NHLBI classification, which focuses primarily on luminal contrast patterns, the IVUS-based scheme enables direct visualization of the vessel wall architecture and more precise delineation of the dissection depth and extent. Consequently, IVUS not only refines the morphological classification of ICAD but also facilitates optimal interventional decision-making by guiding stent placement, detecting residual dissections, and preventing late vessel complications.

### Classification and detection of ICAD by OCT

Under OCT examination, stent-edge dissections can be characterized according to their morphological features, including flap extent, circumferential involvement, and longitudinal length. Definitions and thresholds for clinically relevant stent-edge dissection vary across studies. In ILUMIEN III, a major edge dissection was defined as one involving ≥60° of the vessel circumference or extending ≥3 mm in length (14). In contrast, natural-history data from Radu et al. suggest that small, non-flow-limiting dissections confined to the intima, particularly those involving <45° of the vessel circumference and extending <2 mm in length, generally have a favorable course without additional intervention (15). Furthermore, once classified by size, the dissection can be further characterized by the involved vessel wall layer: intimal dissection (tear limited to the intima), medial dissection (extension into the media, possibly with hematoma), and adventitial (or extramedial) dissection (disruption extending beyond the external elastic membrane into the adventitia) (15).

Compared to angiography-based classifications, the high resolution of OCT allows for more detailed assessment of the flap morphology (e.g., flap angle relative to lumen circumference), depth of vessel wall injury, and the presence of associated intramural hematoma or adventitial disruption—parameters that may influence decisions regarding additional stent coverage or other corrective strategies (16).

### Comparison of different imaging modalities for dissection detection

Although direct head-to-head validation data for ICAD detection are limited, OCT provides substantially higher spatial resolution than IVUS (axial resolution approximately 10–20 μm vs. 100–150 μm), enabling more detailed visualization of subtle vessel-wall abnormalities and dissection morphology (17). In the ILUMIEN III trial, OCT guidance resulted in fewer major stent-edge dissections (flap ≥60° or ≥3 mm), and IVUS detected these events less consistently than OCT (14). The higher spatial resolution of OCT during PCI allows more accurate visualization of side-branch ostia, guidewire position, and stent architecture—including detailed assessment of malapposition. OCT also demonstrates superior ability to identify intimal and medial dissections compared with IVUS, with both modalities outperforming angiography (17). A detailed OCT assessment of the dissection severity was possible, influencing the subsequent management of this complication (18).

Although OCT provides improved resolution for dissection visualization, its need for contrast injection carries a theoretical risk of extending a pre-existing dissection. In addition, most instances of stent malapposition characterized by a short strut–vessel (S–V) distance, as well as findings such as thrombus, tissue prolapse, or small edge dissections, tended to resolve spontaneously on follow-up imaging. These minor abnormalities identified by OCT generally do not necessitate additional intervention (19). Furthermore, in the ILUMIEN III: OPTIMIZE PCI trial, OCT-guided PCI achieved a final minimum stent area that was noninferior to IVUS-guided PCI, with comparable procedural success between the two intravascular imaging strategies (14).

In conclusion, while OCT provides superior spatial resolution for detailed characterization of dissection morphology, its additional anatomical detail may not always translate into a need for further intervention, particularly for small, non-flow-limiting abnormalities. The angiographic classification and characteristic IVUS and OCT findings of procedure-induced coronary artery dissection are summarized schematically in Figure 3.

**Figure 3:**
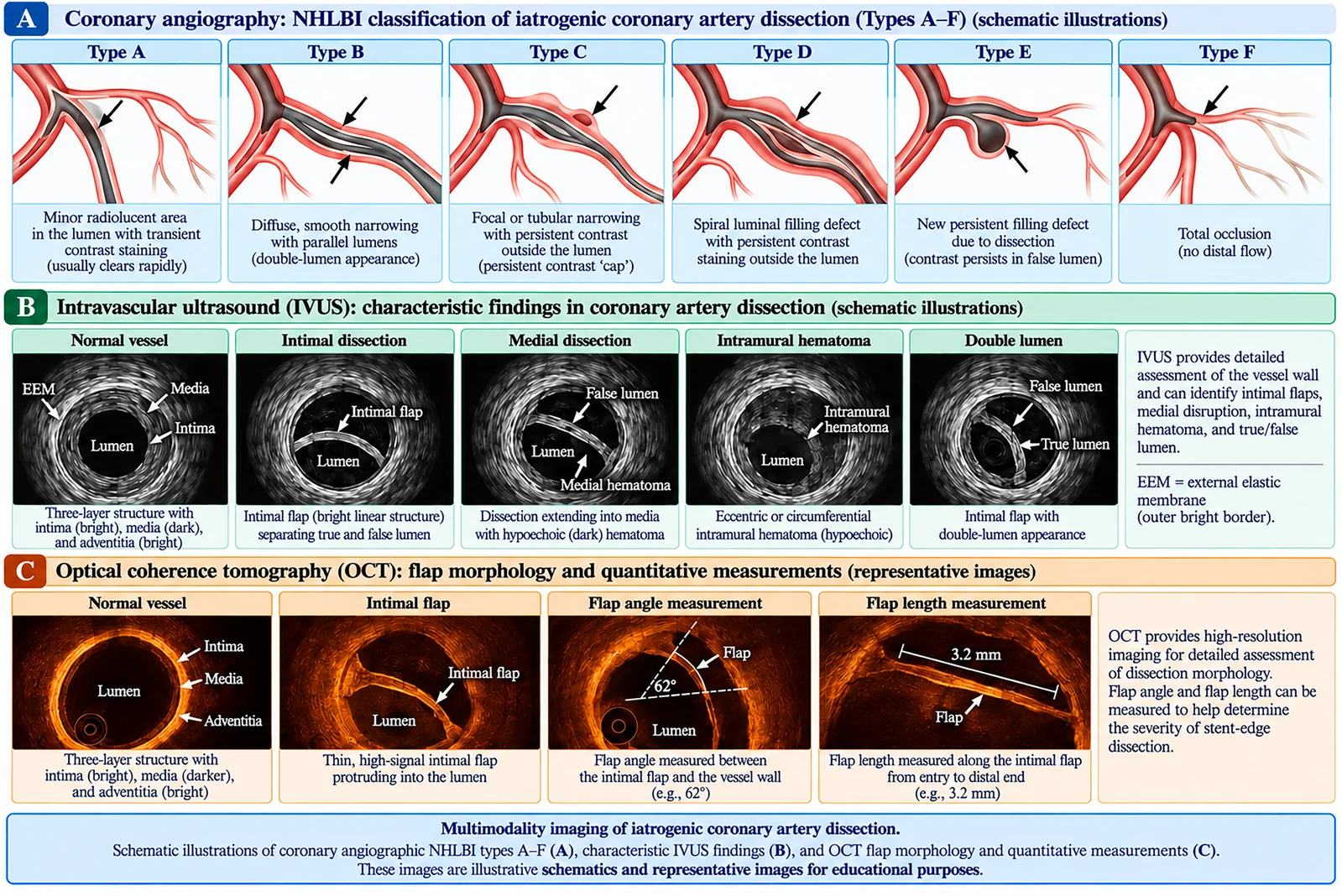
Multimodality imaging of iatrogenic coronary artery dissection. **(A)** Schematic illustration of the National Heart, Lung, and Blood Institute (NHLBI) angiographic classification of coronary dissection (Types A–F). **(B)** Schematic IVUS findings illustrating normal vessel-wall anatomy and characteristic features of coronary dissection, including intimal dissection, medial involvement, intramural hematoma, and a double-lumen appearance. **(C)** Schematic OCT findings illustrating normal vessel-wall anatomy, an intimal flap, and quantitative assessment of dissection morphology by flap angle and flap length. All panels are schematic illustrations created for educational purposes and are not derived from individual patient data. The schematic figure was generated with the assistance of OpenAI's image-generation tool and reviewed by the authors for scientific accuracy. IVUS, intravascular ultrasound; NHLBI, National Heart, Lung, and Blood Institute; OCT, optical coherence tomography.

## Risk factors for ICAD

The development of ICAD is multifactorial, involving the complex interplay of patient characteristics, arterial anatomy, lesion morphology, and procedural techniques. Understanding these risk determinants is critical for both prevention and optimal management during PCI. Overall, the predisposing factors can be broadly categorized into physiological, anatomical, plaque-related, and procedure/device-related domains. Physiological factors, such as female sex and aging, may increase vessel fragility through hormonal or structural vascular changes; anatomical and plaque characteristics determine the susceptibility of specific coronary segments to dissection; and procedural or equipment-related factors, including catheter type, balloon or stent selection, and operator technique, can directly precipitate intimal injury (Figure 4).

**Figure 4:**
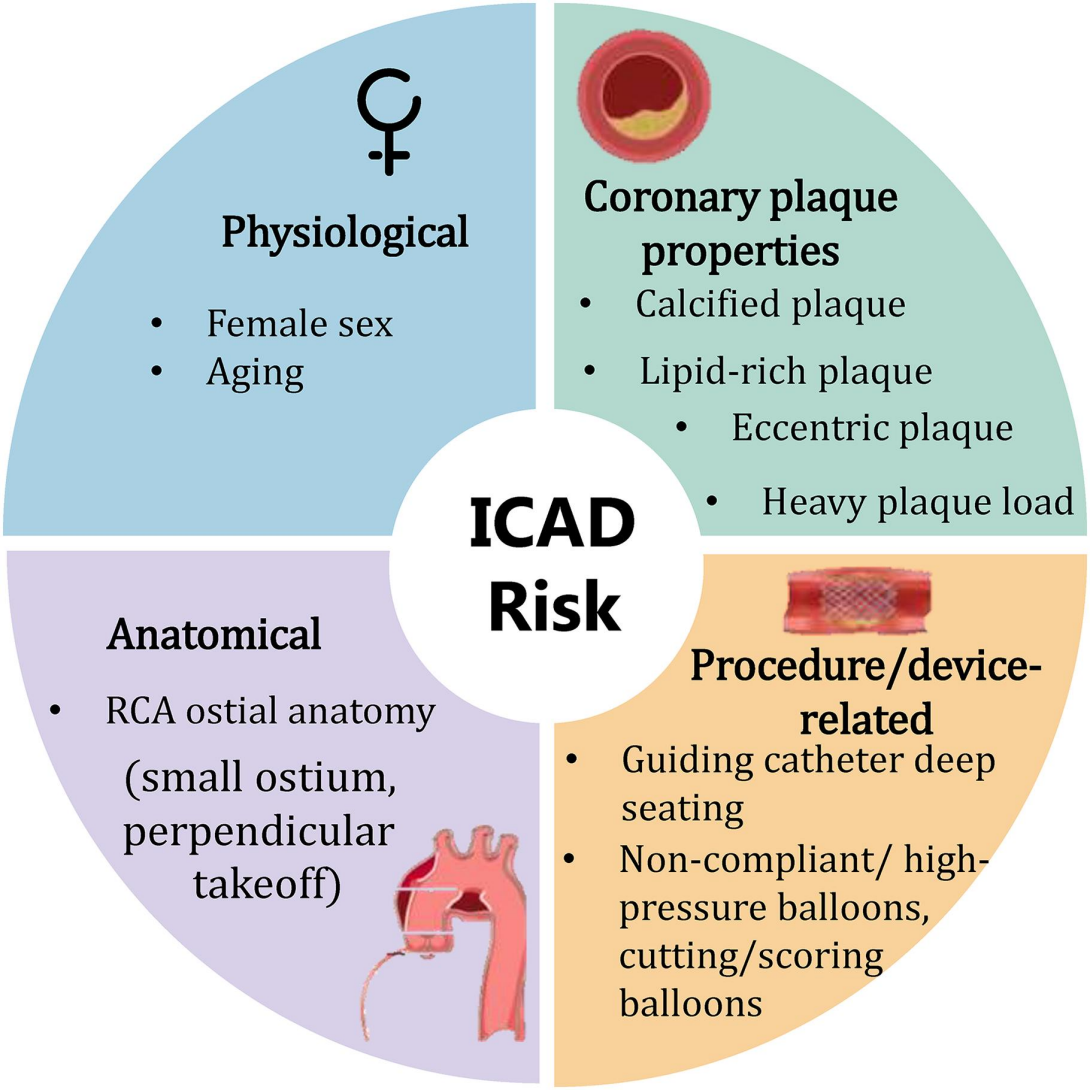
Risk factors contributing to iatrogenic coronary artery dissection (ICAD). Risk factors for ICAD can be broadly categorized into physiological, anatomical, plaque-related, and procedure- or device-related factors. Physiological factors include female sex and aging; anatomical susceptibility is primarily related to right coronary artery ostial anatomy; plaque-related factors include calcified, lipid-rich, eccentric, and high-burden plaques; and procedure- or device-related factors include deep guiding catheter seating and the use of non-compliant or high-pressure, cutting, or scoring balloons. The figure was created by the authors using Microsoft PowerPoint.

### Physiological factors

#### Female sex

Female sex has been consistently associated with an increased risk of ICAD. In the study by Ramasamy et al., 12 of 18 (66%) type F iatrogenic catheter-induced ostial coronary artery dissections occurred in female patients (20). This vulnerability may be partly explained by hormonal and connective-tissue factors, which may influence arterial-wall integrity and increase susceptibility to coronary dissection (21, 22). More directly, a multicenter Japanese registry demonstrated that female sex, complex PCI, and proximal coronary lesions were independent predictors of ICAD. Taken together, these findings suggest that structural and hormonal differences may contribute to the greater susceptibility of female patients to catheter- or device-induced injury (23).

#### Aging

Advancing age may represent another physiological contributor to ICAD susceptibility. With aging, the arterial system undergoes structural and functional remodeling—from large elastic arteries to microcirculatory vessels—leading to reduced elasticity, increased stiffness, and endothelial dysfunction (24). Population-based studies have further demonstrated age-related increases in arterial stiffness and declines in endothelial vasodilator function (25–27). Collectively, these vascular changes may reduce arterial compliance and plausibly increase susceptibility to mechanical injury during catheter manipulation or PCI. Hence, age-related vascular remodeling may provide a physiological substrate that increases vulnerability to procedure-induced coronary injury.

### Anatomical factors

#### Right coronary artery (RCA)

Anatomical predisposition also plays a major role in ICAD. The RCA has been reported as the most frequently affected vessel in catheter-induced dissections (12). This trend likely arises from multiple structural and procedural factors. The RCA ostium is generally smaller and has an almost perpendicular takeoff (60–88°) from the aortic wall, resulting in a less coaxial alignment with catheters—particularly when using a radial approach. Histologically, the RCA ostium contains a denser network of type III collagen fibers and less fibrous reinforcement than the left main coronary artery (LMCA), which may further increase vulnerability to intimal tearing (28). Differences in operator engagement technique may also contribute to this increased risk. In the systematic review by Shorrock et al., the RCA was the most common initial site of dissection (74.8%), followed by the left main coronary artery (15.9%) (29). Similarly, Núñez-Gil et al. reported that iatrogenic aortic dissection occurred while attempting to engage the RCA in 56.8% of cases and the left coronary artery in 40.5% (12). This disparity may relate to structural differences at the arterial ostia, as the LMCA typically exhibits a thicker and more fibrotic ostial wall compared with the RCA (30, 31). Operator technique may also contribute, as the RCA often requires deeper or more aggressive catheter engagement to obtain adequate support, inadvertently raising the risk of dissection.

### Coronary plaque properties

#### Calcified plaque

Severely calcified lesions are still difficult to treat during PCI (32). They often respond suboptimally to PCI and are linked to higher rates of stent underexpansion, restenosis, target lesion revascularization (33), procedural vessel dissection (34), stent-delivery failure (35), and even balloon rupture (36, 37). Efforts to optimize stent expansion and apposition—such as high-pressure post-dilation or the use of calcium-modifying strategies including cutting or scoring balloons, rotational or orbital atherectomy, and intravascular lithotripsy—may inadvertently elevate the risk of ICAD (38, 39). Interestingly, after drug-coated balloon angioplasty, angiographically minor and non-flow-limiting dissections were associated with increased late lumen enlargement at follow-up in intravascular imaging studies (40, 41); whether type C dissections, traditionally classified among higher-grade injuries, genuinely belong in this benign category when they are non-flow-limiting remains unresolved. In the study by Cortese et al. (41), non-flow-limiting dissections included NHLBI types A–C with preserved TIMI grade 3 flow.

#### Attenuated or lipid-rich plaque

Plaques rich in lipids and necrotic core are structurally weak and prone to mechanical deformation during stent expansion. IVUS imaging has shown that edge dissections are associated with adverse plaque characteristics, including calcified or attenuated plaque and greater residual plaque burden (3). Soft plaques on IVUS have been associated with a high lipid content (42). The disruption of a thin fibrous cap and the propagation of mechanical stress along the lipid interface facilitate extension of dissection into adjacent normal segments (43). Consequently, lipid-rich plaques represent not only a marker of vulnerability but also a mechanical weak point during PCI.

#### Eccentric plaques

Eccentric or fibrocalcific plaques create asymmetrical vessel compliance, leading to uneven radial forces during balloon inflation or stent deployment. This mechanical imbalance promotes localized overexpansion and intimal tearing at the plaque edge (44, 45). Recent intravascular imaging and lesion-mechanics analyses have demonstrated that non-uniform lesion geometry significantly alters local wall stress and deformation during PCI, increasing the risk of complications such as edge dissection. Clinically, eccentric fibrocalcific lesions—especially those with high calcium eccentricity—are more prone to vessel injury, dissections, and suboptimal stent expansion (13, 46). Pre-procedural intravascular imaging and calcium modification are therefore essential to minimize the risk of edge dissection in such morphologies.

#### Heavy plaque load

A high plaque burden at or adjacent to the stent edge increases the likelihood of edge dissection (13) and has also been associated with subsequent stent-edge restenosis (47). Data from the ADAPT-DES study demonstrated that lesions with greater plaque burden, calcium content, or attenuated plaque were independent predictors of post-procedural edge dissection (3). From a biomechanical perspective, excessive plaque accumulation limits vessel compliance and leads to abrupt transitions between stented and non-stented segments, facilitating the propagation of dissection flaps. Thin-capped intimal plaques represent mechanically unstable regions prone to initiating and extending dissections, a pattern supported by both histological and imaging evidence of edge disruption (18). These findings underscore the importance of optimal lesion preparation, appropriate stent sizing, and imaging-guided deployment to prevent ICAD in patients with a heavy atherosclerotic burden.

#### CTO lesion

CTOs are among the lesion types most frequently associated with ICAD (48). During antegrade or retrograde dissection and reentry procedures, operators intentionally use the subintimal space to guide the wire toward the true lumen. However, extension of the dissection can jeopardize the aortic ostium in retrograde approaches or compress side branches through subintimal hematoma formation in antegrade strategies (49, 50).

### Procedure- and device-related factors

#### Catheter design and manipulation

A major mechanism leading to dissection is improper catheter positioning or manipulation, often compounded by excessively deep or forceful intubation (51). Catheter selection is an important determinant of catheter-induced ICAD. Evidence from prior studies indicates that guiding catheters carry approximately twice the risk of ICAD compared with diagnostic catheters (12, 52). Several features may account for this increased susceptibility. Guiding catheters often require active intubation maneuvers to achieve adequate support, and their stiffer shafts may transmit greater mechanical force to the coronary ostium. In addition, their design—characterized by a larger internal lumen and enhanced backup to facilitate delivery of balloons and stents—can further increase the likelihood of ostial trauma. Among the various catheter types, Amplatz-shaped catheters demonstrate a particularly strong association with ICAD (12). The manipulations needed to engage the coronaries, coupled with the counterintuitive catheter movement during advancement or withdrawal, may predispose the ostium to injury. This risk is especially pronounced in the RCA, where Amplatz catheters provide excellent support but exert more aggressive force on the ostium. Deep intubation of the guiding catheter during balloon retrieval represents another important mechanism and accounted for 26% of catheter-induced ICAD cases in a previous report (52).

#### Pressure waveform abnormalities

Abnormal pressure waveforms may provide an important warning sign of excessive catheter engagement and impending ostial injury. Two retrospective analyses reported that most LMCA dissections were attributed to suboptimal positioning of either diagnostic or guiding catheter (51, 53). Continuous monitoring of pressure waveforms is essential, as damping or ventricularization indicates excessive engagement of the ostium and an increased likelihood of catheter-induced injury. Additional contributing factors include poor coaxial alignment, overly deep intubation, aggressive catheter shapes, and angioplasty-related mechanical forces acting on the vessel wall.

#### Deep intubation of extension catheters

Although extension catheters enhance support and facilitate device delivery across resistant lesions, they may also increase the risk of coronary dissection. In a consecutive series of GuideLiner-supported PCI, coronary dissection attributed to GuideLiner use occurred in 3.3% (12/363) of cases (54), highlighting the importance of careful manipulation and continuous monitoring during their use.

#### IVI–related risk factors

IVI itself may occasionally contribute to ICAD, particularly when catheter advancement or contrast injection is performed in anatomically vulnerable vessels. IVUS can cause mechanical trauma during catheter advancement or deep engagement, especially in tortuous, calcified, or ostial coronary segments. OCT may pose an additional risk because image acquisition requires transient blood clearance with intracoronary contrast injection. Forceful or high-pressure contrast injection may cause or propagate a coronary dissection, particularly when an intimal injury or dissection is already suspected (55). Therefore, intravascular imaging should be performed with careful catheter manipulation and appropriate contrast injection, and the potential risk of dissection extension should be considered when selecting an imaging modality in patients with suspected ICAD.

#### Polymer-jacketed guidewires

These guidewires improve crossing success in complex lesions such as CTOs, but their smooth coating reduces tactile feedback and increases the risk of subintimal entry or vessel perforation, especially in tortuous or calcified arteries (56, 57). Gentle manipulation and early wire exchange for non-hydrophilic wires after microcatheter delivery are recommended to minimize trauma.

#### Non-compliant or super high-pressure balloons

While these balloons are essential for adequate stent expansion in calcified lesions, oversizing or excessive inflation pressure may produce deep intimal tearing and edge dissections (58). Studies have documented balloon-induced dissections with inappropriate balloon sizing or inflation (59); thus, gradual stepwise inflation and appropriate vessel sizing, including an approximately 1:1 balloon-to-artery ratio, are important (38).

#### Cutting and scoring balloons

These devices create controlled micro-incisions to modify plaque and facilitate expansion, generally reducing uncontrolled tears (60). However, when oversized or overinflated, they may still induce deep medial dissections (58). Controlled inflation under intravascular imaging guidance is advised.

#### Rotational and orbital atherectomy (RA/OA)

RA and OA are valuable for modifying heavily calcified plaques but are associated with procedural complications, including coronary dissection and perforation (61), particularly when the burr-to-artery ratio exceeds 0.6, ablation runs are prolonged, or speed control is poor. Current expert consensus recommends imaging-guided atherectomy with a burr-to-artery ratio between 0.4 and 0.6 (62).

#### Intravascular lithotripsy (IVL)

IVL safely fractures calcium and improves stent expansion with low dissection rates; however, rare balloon ruptures have caused severe dissections (63, 64). Careful pressure monitoring and appropriate balloon sizing remain essential to minimize procedural complications.

#### Drug-coated balloons (DCB)

Most dissections following DCB angioplasty are angiographically minor (Types A–C) and non–flow limiting, with favorable healing and long-term outcomes (65). However, DCB use in long or calcified lesions, particularly in the left anterior descending artery, may increase dissection frequency (66).

### Technique-related risk factors

Technical risk factors include operator experience and clinical decision-making, as well as the selected sizes for the stent, pre-dilation balloon, and post-dilation balloon. Suboptimal catheter handling—such as poor coaxial alignment, deep intubation, or failure to recognize pressure damping—can directly injure the coronary ostium or proximal segments. In addition, improper device selection or use (e.g., choosing an oversized balloon, underestimating lesion calcification, or performing high-pressure dilation without imaging guidance) increases localized wall stress and predisposes to ICAD. Intravascular imaging–guided sizing and careful operator technique may help reduce the risk of these complications (67, 68). The reported quantitative associations between these risk factors and ICAD are summarized in Table 1.

**Table 1:** Quantitative summary of reported risk factors for iatrogenic coronary artery dissection.

Risk factor	Source	Reported effect estimate	Population/notes
Female sex	Hiraide et al. (23)	OR 2.18 (95% CI, 1.53–3.10)	Multicenter Japanese PCI registry
Complex PCI	Hiraide et al. (23)	OR 2.19 (95% CI, 1.58–3.04)	Multicenter Japanese PCI registry
Proximal lesion	Hiraide et al. (23)	OR 1.55 (95% CI, 1.06–2.28)	Multicenter Japanese PCI registry
Guiding vs. diagnostic catheter	Núñez-Gil et al. (12); Eshtehardi et al. (52)	No adjusted estimate reported	∼2-fold higher risk with guiding catheters
RCA vs. LMCA as initial dissection site	Núñez-Gil et al. (12); Shorrock et al. (29)	No adjusted estimate reported	RCA 74.8% in Ref. 29; RCA 56.8% and left coronary 40.5% in Ref. 12
Deep intubation during balloon retrieval	Eshtehardi et al. (52)	No adjusted estimate reported	Reported in 26%
Guide-extension deep intubation	Waterbury et al. (54)	No adjusted estimate reported	Coronary dissection attributed to GuideLiner use occurred in 3.3% (12/363) of cases
Plaque burden/calcium/attenuated plaque	Kobayashi et al. (3)	Calcified plaque at the proximal stent edge: RR = 1.72; *P* = 0.04; attenuated plaque at the distal stent edge: RR = 3.52; *P* = 0.004; distal reference plaque burden: RR = 1.56; *P* < 0.0001	ADAPT-DES IVUS substudy; 2,062 patients (2,433 lesions) undergoing drug-eluting stent implantation

CI, confidence interval; ICAD, iatrogenic coronary artery dissection; IVUS, intravascular ultrasound; LMCA, left main coronary artery; OR, odds ratio; PCI, percutaneous coronary intervention; RCA, right coronary artery; RR, relative risk.

## Features of the dissection that require attention

Evaluating ICAD begins with identifying the involved vessel-wall layer and understanding its anatomical and functional implications. Key features include the location and degree of the dissection, surrounding vascular conditions, associated clinical symptoms, and short-term dynamic changes. These elements guide whether conservative management or immediate intervention is required.

### Degree of dissection

The severity of a dissection can be characterized by its direction, circumferential angle, depth, and length, each of which may influence its clinical significance. Antegrade dissections may compromise distal flow, whereas retrograde extension toward the aortic root carries a higher risk of catastrophic propagation (69). For post-PCI stent-edge dissections, a circumferential angle ≥60° has been used to define major edge dissection (14) and has also been associated with an increased risk of early stent thrombosis in IVUS-based assessments (70). From a mechanistic perspective, deeper extension into the media or adventitia may provide a greater potential space for intramural hematoma propagation and consequent luminal compromise than a superficial intimal tear. Dissection size and length are clinically relevant, as larger and longer OCT-detected stent-edge dissections are more likely to prompt additional intervention, whereas small, non-flow-limiting dissections generally have a favorable natural history (15, 18). Collectively, these features help determine whether a dissection is likely to remain stable or requires immediate repair.

### Location of dissection

Iatrogenic dissection of the LMCA is uncommon but can have serious consequences if not promptly identified. When diagnosed early and treated appropriately, however, both short-term and long-term outcomes are generally favorable. In one large cohort, the incidence of LMCA dissection was 0.07% (38 of 51,452 procedures), occurring nearly twice as often during PCI as during diagnostic coronary angiography (52). In addition, the location of the dissection should be assessed with respect to the proximal, middle, or distal coronary segment; main versus side branch; and proximal versus distal stent edge.

### Surrounding vascular conditions

Surrounding vascular conditions play a pivotal role in determining the stability and clinical relevance of ICAD. The underlying plaque type directly affects vessel compliance and tearing propensity: lipid-rich or attenuated plaques are mechanically weaker and more prone to flap propagation, whereas heavily calcified plaques resist deformation and may cause abrupt hinge points at the transition between stented and non-stented segments (3). Plaque thickness and burden further influence outcomes; large or eccentric plaques narrow the minimal lumen area and increase shear stress, promoting deeper extension of the dissection flap during balloon inflation or stent deployment (44). Additionally, adjacent side branches are critical anatomical considerations: dissections near bifurcations can alter flow patterns, lead to carina shift, or compress the side-branch ostium through intramural hematoma expansion, thereby increasing the risk of ischemia or a reduced Thrombolysis in Myocardial Infarction (TIMI) flow grade (71). Collectively, these vessel-level features help predict whether a dissection will remain localized and benign or progress to clinically significant obstruction.

### Clinical symptoms

Clinical symptoms provide an immediate functional assessment of ICAD. A reduction in TIMI blood flow grade may indicate luminal compromise from an intimal flap or intramural hematoma, signaling the need for urgent intervention. Patients may develop new or worsening ischemic manifestations, including electrocardiographic ST-segment changes or hemodynamic instability, which reflect impaired distal perfusion. Chest tightness or pain, although nonspecific, often correlates with dynamic luminal obstruction and should prompt reassessment of the dissection morphology (23, 72). Together, these clinical indicators help determine whether conservative observation is acceptable or whether prompt revascularization is required.

### Dynamic changes of dissection

We suggest observing the vessel for 10–15 min after the initial injury to assess the short-term evolution of the dissection. During this interval, the operator evaluates whether the flap extends longitudinally, penetrates into deeper layers, or is accompanied by progressive intramural hematoma formation—changes that may compromise the lumen or worsen TIMI flow (23). Stable dissections remain unchanged during observation, whereas those that enlarge or deepen are more likely to require immediate stent implantation.

## Prognosis, prevention, and management of ICAD

### Prognosis of ICAD

Ramasamy et al. screened 55,968 coronary angiograms over 10 years at Barts Health NHS Trust and identified 51 cases of catheter-induced ostial coronary artery dissection, corresponding to a prevalence of 0.09%, with 3 in-hospital deaths (5.9%) and no additional mortality during a median follow-up of 3.6 years (20). Eshtehardi et al. identified 38 cases of iatrogenic LMCA dissection among 51,452 coronary procedures (0.07%) and reported their 5-year follow-up data, 31 of whom underwent stent implantation and/or CABG; no differences were found regarding death, major adverse cardiac events (MACE), or target vessel revascularization between the two strategies (52). Verevkin et al. described 168 patients who required emergency cardiac surgery for PCI-related iatrogenic injuries, with ICAD being the predominant complication, accounting for 75% of cases (73). The 30-day mortality rate was 20.8%, whereas long-term survival was 71.8% at 1 year, 63.9% at 5 years, 49.6% at 10 years, and 44.6% at 12 years. In another series, Cheng et al. evaluated 13 patients with severe LMCA dissection (types C–F) treated predominantly with PCI; emergency CABG was required in only two patients in whom flow could not be restored percutaneously. Among the 11 patients who underwent successful bailout stenting, no deaths or recurrent myocardial infarctions were observed during a mean follow-up of 30.1 ± 11.8 months (51). Additionally, data from a multicenter Japanese registry demonstrated that catheter-induced coronary artery dissection (CICAD) was associated with a higher incidence of in-hospital adverse events—including new-onset cardiogenic shock and heart failure—irrespective of whether post-procedural flow was impaired, with adjusted ORs of 10.9 (95% CI, 5.30–22.6) for flow-impaired CICAD and 2.27 (95% CI, 1.20–4.27) for flow-recovered CICAD (23). Key cohorts informing the prognosis of ICAD are summarized in Table 2.

**Table 2:** Key cohorts informing ICAD prognosis.

Study/reference	Design/population	Key results
Ramasamy et al. (20)	55,968 coronary angiograms over 10 years	51 catheter-induced ostial coronary artery dissection cases; prevalence 0.09%; 3 in-hospital deaths (5.9%); no additional mortality over median 3.6 years
Eshtehardi et al. (52)	51,452 procedures	LMCA dissection 0.07% (38 cases); ∼2× more frequent during PCI than diagnostic angiography
Verevkin et al. (73)	168 patients, emergency surgery for PCI-related injury	ICAD 75% of injuries; 30-day mortality 20.8%; survival 71.8 ± 4% (1 y), 63.9 ± 4% (5 y), 49.6 ± 5% (10 y), 44.6 ± 6% (12 y)
Cheng et al. (51)	13 patients, LMCA dissection types C–F	11 bailout stenting, 2 emergency CABG; 0 deaths/recurrent MI over 30.1 ± 11.8 months
Hiraide et al. (23)	Multicenter Japanese PCI registry	Higher in-hospital adverse events irrespective of post-procedural flow; adjusted OR 10.9 (95% CI, 5.30–22.6) for flow-impaired CICAD and 2.27 (95% CI, 1.20–4.27) for flow-recovered

CABG, coronary artery bypass grafting; CI, confidence interval; CICAD, catheter-induced coronary artery dissection; ICAD, iatrogenic coronary artery dissection; LMCA, left main coronary artery; MI, myocardial infarction; OR, odds ratio; PCI, percutaneous coronary intervention.

### Prevention of ICAD

Although ICAD cannot be entirely avoided, awareness of patient-, device-, and procedure-related risk factors can help reduce its occurrence during coronary interventions. Continuous assessment of the pressure waveform is essential. Contrast injection should be withheld when damping or ventricularization is observed, and catheter alignment should be adjusted to prevent inadvertent injection into the vessel wall. Persistent pressure damping may also indicate the presence of aorto-ostial stenosis, which may increase vulnerability to ostial injury or dissection (74). Concerns have been raised regarding automatic contrast injection systems because of their fixed high-flow and high-pressure delivery, which reduces operator control and may heighten the risk of vessel wall injury. Although direct comparative data with manual injection are lacking, careful adjustment of machine parameters is advised (75). Suggested injection settings for automated contrast injectors include a flow rate of 3 mL/s with 5 mL of contrast for the RCA and 3–5 mL/s with 6–10 mL for the left coronary artery (75). Catheter morphology also plays a key role in preventing ICAD. Amplatz-shaped catheters, for example, provide strong backup support but exert greater mechanical force at the coronary ostium, resulting in a higher dissection risk than softer, more forgiving designs such as Judkins catheters. Attention to appropriate catheter selection and manipulation therefore remains a fundamental preventive measure.

The CLI-OPCI II study showed that dissections >200 µm at the distal stent edge detected by OCT were independently associated with MACE (HR: 2.54; *P* = 0.004) (76). Small edge dissections identified on OCT but not visualized on angiography are generally considered clinically insignificant (15, 18, 77, 78). However, several morphological characteristics should be evaluated, including the longitudinal and circumferential extent of the tear and the depth of involvement across the intima, media, or adventitia.

### Management of ICAD

When catheter-induced ICAD is identified, the cause and morphological characteristics of the dissection should be assessed, and targeted treatment selected according to the patient's clinical manifestations. Applying the context-specific criteria reviewed above and summarized in Table 3, management should be individualized according to dissection severity, coronary flow, and the patient's clinical status.

**Table 3:** Published criteria relevant to the assessment and management of iatrogenic coronary artery dissection.

Study/reference	Assessment feature	Criterion	Clinical relevance
NHLBI classification (10)	Angiographic dissection severity	NHLBI types C–F	Associated with a higher risk of acute complications; intervention should be considered according to coronary flow and clinical status
ILUMIEN III (14)	OCT-detected major edge dissection	Circumferential involvement ≥60° or longitudinal length ≥3 mm	Classified as major edge dissection
Radu et al. (15)	Small non-flow-limiting edge dissection	Intimal-only, <45° circumferential involvement and <2 mm length	Generally favorable natural history; additional intervention may not be required
CLI-OPCI II (76)	OCT-detected distal stent-edge dissection	Distal dissection >200 μm	Associated with increased risk of adverse cardiac events

OCT, optical coherence tomography.

The immediate priority is to cease contrast injection, as continued injection may exacerbate propagation of the flap. The need for further treatment depends on several morphological features—including the depth, extent, location, flap angle, involvement of inflow or outflow segments, and the remaining effective lumen area (3). Coronary stent implantation to seal the intimal tear, stabilize the vessel, and prevent further dissection extension is the preferred approach for moderate-to-severe ICAD. Reentry strategies, such as using a cutting balloon or aspirating the false lumen through a microcatheter, have been described but are usually facilitated by intravascular imaging guidance (79–81) Conservative management, consisting mainly of beta-blockers and appropriate antiplatelet therapy, may be appropriate for patients who remain asymptomatic, exhibit no electrical or hemodynamic abnormalities, and demonstrate limited, stable dissections after a brief period of observation (corresponding to NHLBI types A and B) (82). A recent case report described successful conservative management of a type B ICAD in a patient without chest pain, preserved distal coronary flow, and no electrocardiographic changes, with complete angiographic healing observed 45 days after the procedure (83). Evidence for this approach, however, is based largely on case reports and observational studies (84). In contrast, severe dissections (i.e., NHLBI types C to F) or compromised flow should be considered for PCI (82, 85), although CABG has also been described as a potential therapeutic strategy (86, 87), especially when true lumen wiring cannot be achieved (73).

Special consideration is required when the dissection extends into the aortic root or ascending aorta. The extent of aortic involvement alone should not be regarded as an absolute indication for surgery. The primary therapeutic objective is to seal the entry tear, restore or preserve true-lumen and coronary blood flow, and prevent further propagation of the dissection (84). Stable patients with limited aortic extension may be managed conservatively with close imaging surveillance, whereas PCI should be considered when the entry tear can be safely sealed. Surgical consultation should be considered for persistent or worsening chest pain, hemodynamic instability, progressive dissection, severe aortic regurgitation, aortic valve or arch involvement, or cardiac tamponade, while emergency surgery is generally reserved for failed percutaneous sealing or threatened vital aortic branches (72, 88). Cardiac CT angiography is useful for defining residual dissection and follow-up (89) (Figure 5).

**Figure 5:**
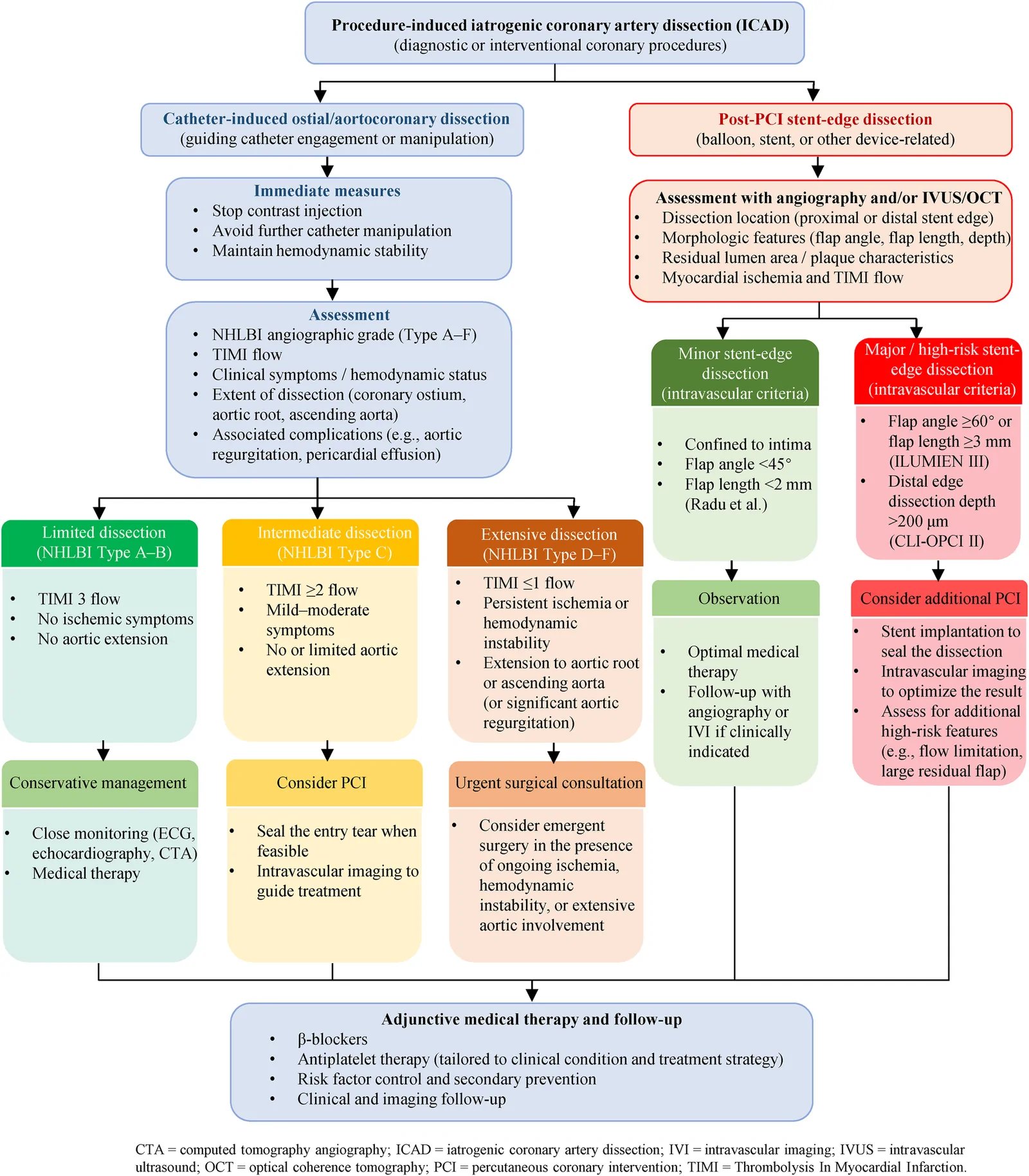
Proposed management framework for procedure-induced iatrogenic coronary artery dissection (ICAD). Management should be individualized according to the mechanism and anatomical context of the dissection. For catheter-induced ostial or aortocoronary dissection, treatment decisions are guided primarily by angiographic severity (NHLBI grade), TIMI flow, clinical and hemodynamic status, and the presence and extent of aortic involvement. For post-PCI stent-edge dissection, IVUS/OCT-derived morphological criteria may help distinguish minor dissections suitable for observation from major or high-risk dissections that may require additional intervention. The OCT-based thresholds summarized in Table 3 were derived from post-PCI stent-edge dissection studies and should not be considered validated thresholds for catheter-induced ostial or aortocoronary dissection. The figure was created by the authors using Microsoft PowerPoint.

## Limitations

Several limitations of this review should be acknowledged. First, as a narrative review, it lacks a predefined systematic protocol, and the literature search was not intended to be exhaustive. Our search was restricted to English-language publications from database inception through 26 August 2026; non-English reports and very recent publications may have been missed, and selective citation cannot be excluded. Furthermore, the evidence base for ICAD is heterogeneous and is largely derived from observational studies, retrospective cohorts, and case reports, with limited high-quality randomized controlled trials. This limits direct comparisons among conservative management, PCI, and CABG, making it difficult to establish a definitive optimal treatment strategy. Therefore, our proposed diagnostic and management framework represents a synthesis of the currently available evidence and should be considered a practical clinical framework rather than a formal guideline or validated treatment algorithm.

## Conclusions

ICAD is an infrequent but clinically significant complication of CAG and PCI. Its occurrence reflects a combination of patient-related susceptibility, plaque morphology, vascular anatomy, and device- or technique-dependent factors. Intravascular imaging has improved diagnostic accuracy and clarified which dissections are benign and which require intervention. However, standardized definitions, optimal management thresholds, and long-term outcome data remain limited. In practice, this translates to continuous pressure-waveform vigilance during catheter engagement, low-threshold intravascular imaging when dissection is suspected, and threshold-based decisions to observe, stent, or consult surgery as depicted in Figure 5. In particular, prospective registries with standardized OCT-based dissection definitions and randomized comparisons of conservative watchful observation versus bailout stenting for non-flow-limiting type B–C dissections are needed.
